# Relative impact of indels versus SNPs on complex disease

**DOI:** 10.1002/gepi.22175

**Published:** 2018-11-22

**Authors:** Sarah A. Gagliano, Sebanti Sengupta, Carlo Sidore, Andrea Maschio, Francesco Cucca, David Schlessinger, Gonçalo R. Abecasis

**Affiliations:** ^1^ Center for Statistical Genetics, and Department of Biostatistics, University of Michigan Ann Arbor Michigan; ^2^ Istituto di Ricerca Genetica e Biomedica, Consiglio Nazionale delle Ricerche (CNR) Cagliari Italy; ^3^ Dipartimento di Scienze Biomediche Università degli Studi di Sassari Sassari Italy; ^4^ Laboratory of Genetics, National Institute on Aging, US National Institutes of Health Baltimore Maryland

**Keywords:** complex traits, genome‐wide association, insertions and deletions (indels)

## Abstract

It is unclear whether insertions and deletions (indels) are more likely to influence complex traits than abundant single‐nucleotide polymorphisms (SNPs). We sought to understand which category of variation is more likely to impact health. Using the SardiNIA study as an exemplar, we characterized 478,876 common indels and 8,246,244 common SNPs in up to 5,949 well‐phenotyped individuals from an isolated valley in Sardinia. We assessed association between 120 traits, resulting in 89 nonoverlapping‐associated loci.We evaluated whether indels were enriched among credible sets of potential causal variants. These credible sets included 1,319 SNPs and 88 indels. We did not find indels to be significantly enriched. Indels were the most likely causal variant in seven loci, including one locus associated with monocyte count where an indel with causality and mechanism previously demonstrated (rs200748895:TGCTG/T) had a 0.999 posterior probability. Overall, our results show a very modest and nonsignificant enrichment for common indels in associated loci.

## 1 INTRODUCTION

The relative impact of insertion and deletion variants (indels) and single‐nucleotide polymorphisms (SNPs) on human complex disease risk is unclear. By definition, an SNP changes a single nucleotide in the DNA sequence, whereas an indel incorporates or removes one or more nucleotides (Loewe, 2008).

SNPs in coding and noncoding regions have been implicated in both Mendelian and complex disease, and the same is true for indels. In coding regions, an insertion or deletion that is not in‐frame (a multiple of three base pairs) will alter the reading frame resulting in a new set of amino acids and thus a protein product that differs to the wild type. The presence of 40 or more cytosine adenine guanine (CAG) repeats in the first exon of the huntingtin gene (*HTT*) results in Huntington's disease (Lench et al., 2013). Even in‐frame indels (insertions or deletions of three or multiples of three base pairs) in the coding sequence can also result in altered proteins. An example is a deletion in the cystic fibrosis transmembrane conductance regulator (*CFTR*) gene that removes one amino acid (phenylalanine) at position 508, which arrests protein function and leads to cystic fibrosis (Mullaney, Mills, Pittard, & Devine, 2010). With regard to noncoding regions, as with SNPs, indels may have effects on the affinity of a binding site for a regulatory factor or transcriptional machinery, or on chromatin structure. For example, insertions within the promoter region of the *FMR1* gene can cause Fragile X syndrome (Mills et al., 2006), and insertions within the promoter region of the *SNCA* gene contributes to autosomal dominant Parkinson's disease (Singleton et al., 2013).

There is a balance between mutation rates and selective constraint of indels, particularly in the coding sequence as reading frames should be maintained and thus the protein function preserved. Coding indels tend to be subject to stronger purifying selection than SNPs (de la Chaux, Messer, & Arndt, 2007, Montgomery et al., 2013).

The cumulative contribution of indels compared with SNPs to disease risk has not been thoroughly investigated. To fill this gap in our understanding, we assessed associations of indels and SNPs with 120 traits in a sample of up to 5,949 individuals from the island of Sardinia. We limited our analysis to common variants because of the nature of the study design. Acknowledging that this work lacks insight from rare variation, it nevertheless begins to move toward a better understanding of which type of polymorphism is more likely to impact human health and to quantify the gain by routinely including indels in genome wide association studies (GWAS).

## 2 RESULTS

### 2.1 Imputation of indels

A total of 928,605 of 1,156,646 autosomal indels remained after imputation quality (RSQR) thresholds were applied, and they are distributed throughout the autosomes (Supporting Information Figure S1). With regard to autosomal SNPs, 17,607,889 of 24,106,694 passed the RSQR thresholds. Applying a minor allele frequency (MAF) ≥ 1% cutoff to ensure the inclusion of variants with high imputation quality, there were 8,725,120 variants genome wide (478,876 indels and 8,246,244 SNPs). All downstream analyses involve this filtered set of variants.

Imputation quality summary metrics are displayed in Supporting Information Figure S2.

### 2.2 Annotation of indels

The vast majority of indels are noncoding, and only 0.2% (760) of the MAF ≥ 1% indels fall into a coding region as defined by GENCODE v19 (Harrow et al., 2012). A total of 58% of the indels are deletions and 42% are insertions (276,508 and 202,367, respectively). This inequality in proportions is likely because of additional challenges aligning reads containing insertions larger than the fragment size in the sequencing library (Medvedev, Stanciu, & Brudno, 2009).

There were fewer indels in coding regions compared with noncoding regions than expected by chance (Table 1). This lower relative density of indels in the coding region has been seen previously in other datasets (Lek et al., 2016; Mullaney et al., 2010).

**Table 1:** Numbers of coding and noncoding variation (MAF ≥ 1%) in SardiNIA

	Coding	Noncoding	Total
SNPs	60,844 (0.7% of SNPs)	8,185,400 (99.3% of SNPs)	8,246,244 (95% of variants)
Indels	**760** (0.2% of Indels)	478,116 (99.8% of indels)	478,876 (5% of variants)
Total	83,223	8,641,897	8,725,120

*Note*. Bold value indicate *p* < 2.2E−16. SNPs: single‐nucleotide polymorphisms.

A total of 56% of the indels are the insertion or deletion of a single base (148,355 deletions and 120,098 insertions).

We assessed the proportion of SNPs and indels within 1Mbp of associated loci (see Table 2) in regions of low complexity (amino acid sequences that contain repeats of single amino acids or short amino acid motifs making these regions more difficult to call) (Morgulis, Gertz, Schäffer, & Agarwala, 2006). These regions represent 8.8% of the whole‐genome autosomal sequences (254,665,411 base pairs). There were significantly more indels than SNPs found in these regions (chi‐square = 1441.1; *p* = 2.5E−315), likely because of high error rate in variant calling. Of the 18,325 indels found within 1Mbp of associated loci, we detected 4.3% (792) to be in regions of low complexity. Of the 308,310 SNPs found within 1Mbp of associated loci, we detected 1.1% (3,354) to be in regions of low complexity.

**Table 2:** Enrichment results for indels and SNPs in associated loci and for controls

Category	Percentage within 1Mbp associated loci	Enrichment parameter, *λ* (*p*)
Indel versus SNP	3.8% (18,325) versus 3.7% (308,310)	0.09 (0.88)
Missense versus not	7.6% (2,356) versus 3.7% (324,279)	3.92 (1.6E−10)
Coding Indel versus not	8.0% (61) versus 3.7% (326,574)	(−16.3) (0.90)

*Note*. SNPs: single‐nucleotide polymorphisms.

A total of 6.3% of indels (30,124) were not found in the variant list of the NHLBI Trans‐Omics for Precision Medicine (TOPMed) high‐depth whole‐genome sequencing effort.

### 2.3 Association analyses

Using the MAF ≥ 1% cutoff, 51 of the 120 traits tested had at least 1 variant that reached genome‐wide significance (*p* ≤ 5E−8), and for 33 of those 51 traits, at least 1 indel reached genome‐wide significance. These association results allowed us to assess the relative enrichment of indels and SNPs among trait‐associated variants. There were 9,474 variants that reached genome‐wide significance, of which 494 are indels, in at least 1 of the traits tested for association.

Of the significant indels, 19 are not found in the TOPMed variant list.

### 2.4 Impact of indels versus SNPs

We investigated whether indels are more likely than SNPs to be potentially causal. To obtain an estimate of indel enrichment among potentially causal variants, we assessed the proportion of indels to SNPs within 1Mbp of associated loci (*N* = 89) compared with the rest of the genome for variants MAF ≥ 1%. We set a wide base pair range to ensure that all possible causal variants would be included in the computation of credible sets (see below) regardless of the linkage disequilibrium structure at the loci. Indels were not significantly enriched (estimate = *e* ^(0.09)^ = 1.09; *p* = 0.88).

To address the inherent genomic alignment and calling challenges in regions of low complexity, we removed SNPs and indels that fall into those regions and then repeated the analysis to estimate the enrichment parameter. Four percent of indels (*N* = 792) in the associated loci were removed, and 1% of SNPs (*N* = 3,354) were removed. The estimated enrichment parameter remained nonsignificant (estimate = 1.41, *p* = 0.64).

We obtained 95% credible sets of potentially causal variants. Of the variants in the credible sets, the distribution of effect sizes did not significantly differ between indels and SNPs (the Mann‐Whitney *U* test *p* = 0.91). Indels were the most likely causal variant in 7 of the 89 associated loci assessed. One of those sets contained only one variant, solely an indel in the 3′‐untranslated region of the *TNFSF13B* gene (rs200748895:TGCTG/T; chi‐square = 24.9; posterior probability = 0.999) for association with monocyte count. This complex polymorphism has been identified as the causal variant at this locus in previous work (Steri et al., 2017). Thus, the identification of a known causal variant provided us with reassurance of the utility of our method. Of variants with a posterior probability ≥0.1, 8% (14/182) were indels. Although 8% of indels with a posterior probability ≥0.1 within the credible sets is not more than expected by chance (chi‐square = 0.74; *p* = 0.39), this percentage nevertheless is higher than the proportion of indels in the total number of variants.

As a positive control for the enrichment parameter, we assessed whether missense SNPs are enriched in trait‐associated loci given their direct consequence on the amino acid chain and thus the resulting protein. The estimated enrichment parameter showed that missense SNPs are more likely to be potentially causal than other variants (estimate = *e* ^(3.92)^ = 50.4; *p* = 1.6E−10) (Table 2). Relatively there are more indels than missense SNPs in the genome, and thus the nonsignificant enrichment results for the indel versus SNP analysis is unlikely because of a lack of power possibly a result of the lower imputation quality in indels compared with SNPs. Genome wide, there were 31,112 missense SNPs with MAF ≥ 1% in the data set, of which 7.6% (2,356) fell into trait‐associated loci.

As a complementary analysis to the missense analysis, we also performed a coding indel enrichment analysis. Of the 760 indels in the data set with MAF ≥ 1% that fall into coding sequences, 8.0% (61) were in trait‐associated loci. The estimated enrichment parameter showed that coding indels are not more likely to be potentially causal than other variants (estimate = *e* ^(−16.3)^ = 8.3E−8; *p* = 0.90) (Table 2). However, we acknowledge the lack of power in this particular subanalysis of a small subset of variation.

## 3 DISCUSSION

Using association results from the SardiNIA cohort of up to 5,949 individuals for 120 traits, we did not find evidence of common indels more likely to be potentially causal than SNPs with regard to associations to complex traits. On a similar note but looking at only the coding sequence, Montgomery et al. (2013) did not find direct evidence that potentially causal classes of coding indels are enriched for associations compared with known disease‐associated SNPs present in the GWAS Catalog.

The modest sample size in our study limits the capacity to identify causal variants. However, our analysis strategy allowed us to evaluate the enrichment of indels at loci even in situations where we could not pinpoint an individual causal variant, which may require studies with larger sample sizes or multiple ancestries. We also acknowledge that a subset of variants achieving the widely accepted genome‐wide significant *p* value threshold (*p* ≤ 5E−8) could be false positive signals. In addition, we applied a MAF threshold to ensure the integrity of the imputed genotypes, but in doing so we removed potentially causal rare variants, possibly biasing our analysis. Future studies with larger sample size will help in addressing these limitations by increasing the statistical power. Finally, in vitro and in vivo experimental designs are required to verify the functionality of the variants in question. We employ an in silico method to address potential “causality,” which can guide the choice of variants to carry forward to these subsequent experiments.

Investigation into the relative impact of common and also lower frequency indels compared with SNPs in the context of larger more diverse samples and more phenotypes is warranted.

## 4 METHODS

### 4.1 SardiNIA study data set

In brief, we genotyped 6,602 individuals from four villages in the Lanusei valley on Sardinia (>60% of the adult population). Each sample was genotyped on four different Illumina Infinium arrays: OmniExpress, Cardio‐Metabochip (Voight et al., 2012), Immunochip (Parkes, Cortes, van Heel, & Brown, 2013), and Exome Chip. We also performed low‐depth (~4× coverage) whole‐genome sequencing on 3,839 individuals, 2,340 of whom we also genotyped. Study samples, genotyping, sequencing, and variant calling have been previously described (Sidore et al., 2015).

More than 100 traits (e.g., blood lab measurements, anthropometric values) have been measured at 4–5 time‐points. We looked at 120 traits from the first visit, for which the majority of the individuals have measurements (median number of samples with at least one measurement per trait = 5,814, first quartile = 5,473, third quartile = 5,923). The traits have been previously summarized (Pilia et al., 2006).

### 4.2 Imputation

We imputed autosomal SNPs and indels for the individuals who were successfully genotyped on all four arrays (*N* = 6,602) using Minimac3 (Das et al., 2016) and the Sardinia sequencing data as the reference panel with genomic locations corresponding to GRCh37. This sequencing panel has 1,156,646 biallelic indels and 24,106,694 biallelic SNPs. We imputed SNPs based on only the phased SNPs in the reference panel (i.e., indels removed), and then we imputed indels based on both the SNPs and indels in the reference panel.

For very rare SNPs (i.e., MAF < 0.5%) we have shown (data not published) that imputation with a Sardinian reference panel outperforms imputation with the reference panel of the Haplotype Reference Consortium (HRC) (McCarthy et al., 2016) samples, which includes 3,445 Sardinian individuals.

After imputation, we retained only markers with an imputation quality (RSQR) > 0.3 or > 0.6 if the estimated MAF was ≥1% or <1%, respectively (Pistis et al., 2015).

### 4.3 Annotation of indels

The likely functional impact of variants was annotated using the Ensembl Variant Effect Predictor (McLaren et al., 2010) to annotate consequences. VT (Tan, Abecasis, & Kang, 2015) was used to annotate DUST low complexity regions (Morgulis et al., 2006). We also looked at the MAF distributions and the lengths of the indels.

We identified which indels were not found in any of the populations in the NHLBI TOPMed release 3a variant list (NHLBI TOPMed Project Freeze 3a. https://www.nhlbiwgs.org). The 3a release contains 170 million variants on 14,559 individuals and was accessed through the BRAVO browser (https://bravo.sph.umich.edu). Indels were considered present in the TOPMed Project if there was an exact match by chromosome, start position, reference allele, and alternate allele.

### 4.4 Association analyses

Association analyses were performed for 120 quantitative traits measured in 1,460–5,949 individuals (median = 5,814) from Visit 1 of the SardiNIA cohort. Associations were run in EPACTS (Kang, Zhan, Sim, Ma, 2012) using the age, age^2^, and sex‐adjusted inverse‐normalized residuals of the outcomes as input to the Efficient Mixed Model Association Expedited (Kang et al., 2010) single variant test (i.e., a linear model with a kinship matrix).

### 4.5 Impact of indels versus SNPs

**Figure 1:**
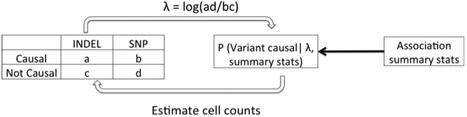
Iterative procedure to estimate enrichment parameter, *λ*

To obtain an estimate of indel enrichment among potentially causal variants, we assessed the proportion of indels to SNPs within 1Mbp of associated loci compared with the rest of the genome. We used a filter of MAF ≥ 1% and estimated an enrichment parameter, which denotes how much more likely indels are potentially causal compared with SNPs. This iterative procedure of essentially maximizing a log odds ratio of a two‐by‐two table to obtain the enrichment parameter is summarized in Figure 1. If this estimate was statistically significant, we would use it to calculate the posterior probability of each variant being causal. To illustrate, say an SNP and an indel have similar *p* values, but the enrichment parameter suggests the enrichment of indels at associated loci. The indel would thus receive a higher posterior probability.

For the traits, we identified all of the variants (MAF ≥ 1%) with a significant GWAS *p* value (*p* ≤ 5E−8). We took 500 K base pairs downstream and 500 K base pairs upstream the most significant variant to obtain the locus. For any overlapping loci, within a trait or among traits, we retained the locus with the most significant *p* value and dropped the other loci. Then, we obtained all of the variants (MAF ≥ 1%) within the nonoverlapping 1Mbp loci and annotated them with regard to being an SNP or an indel.

As a positive control, for the same nonoverlapping 1Mbp associated loci used for the indel versus SNP analysis, we reannotated the variants, this time with regard to being a either a missense SNP or not a missense SNP or an indel. As a complementary analysis, we also reannotated the variants with regard to being a coding indel or not, where coding regions were defined by GENCODE v19.

## CONFLICTS OF INTERESTS

The authors declare that they have no conflicts of interest.

## Supporting information

Supporting information
