# Supplementary material for: Relative impact of indels versus SNPs on complex disease
Source: Genet Epidemiol. 2018 Nov 22;43(1):112–7. doi: 10.1002/gepi.22175 (PMC6330128; doi:10.1002/gepi.22175)
Supplement: Supplementary file 1 — Supporting information [file GEPI-43-112-s001.pdf]

Gagliano et al., 2018. Relative impact of indels versus SNPs on complex disease.

SUPPLEMENTARY MATERIALS

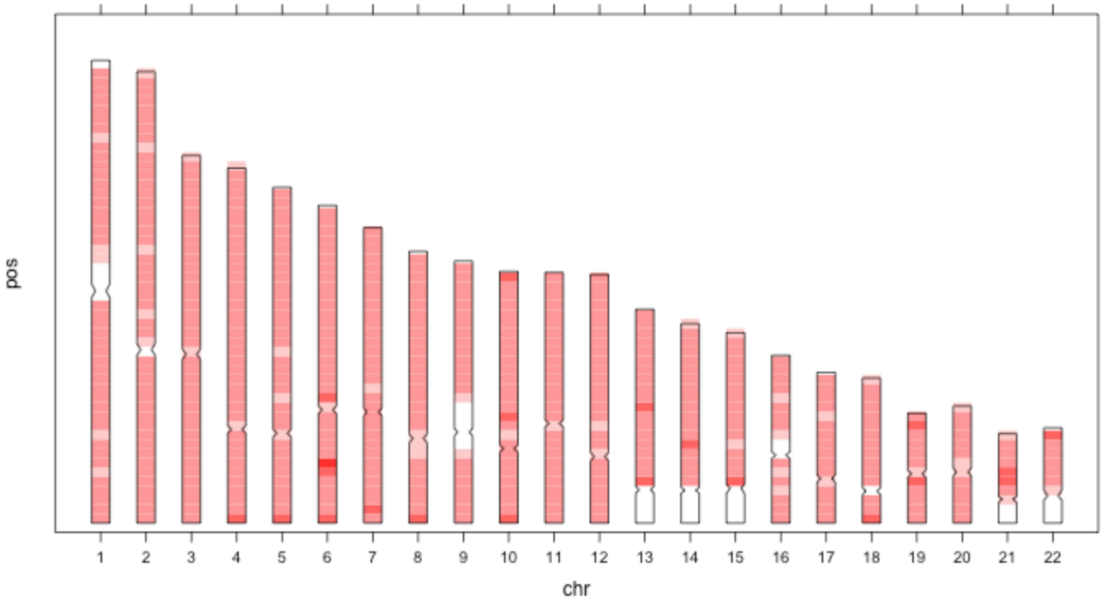

Figure S1. Distribution of indels throughout the autosomes. The darker the colour, the more indels present in that region.

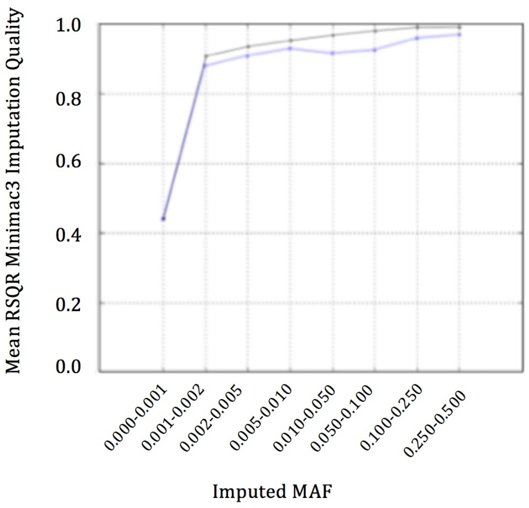

Figure S2. Estimated overall imputation quality metric from minimac3 (RSQR) for SNPs and indels imputed in the SardiNIA cohort for various minor allele frequency (MAF) bins. Black line= SNPs; blue line= Indels
